# Enhanced Performance by Time-Frequency-Phase Feature for EEG-Based BCI Systems

**DOI:** 10.1155/2014/420561

**Published:** 2014-06-17

**Authors:** Baolei Xu, Yunfa Fu, Gang Shi, Xuxian Yin, Zhidong Wang, Hongyi Li, Changhao Jiang

**Affiliations:** ^1^State Key Laboratory of Robotics, Shenyang Institute of Automation (SIA), Chinese Academy of Sciences (CAS), Shenyang 110016, China; ^2^University of Chinese Academy of Sciences, Beijing 100049, China; ^3^School of Automation and Information Engineering, Kunming University of Science and Technology, Kunming 650500, China; ^4^Department of Advanced Robotics, Chiba Institute of Technology, Chiba 2750016, Japan; ^5^School of Mechanical Engineering & Automation, Northeastern University, Shenyang 110004, China; ^6^Key Laboratory of Motor and Brain Imaging, Capital Institute of Physical Education, Beijing 100088, China

## Abstract

We introduce a new motor parameter imagery paradigm using clench speed and clench force motor imagery. The time-frequency-phase features are extracted from mu rhythm and beta rhythms, and the features are optimized using three process methods: no-scaled feature using “MIFS” feature selection criterion, scaled feature using “MIFS” feature selection criterion, and scaled feature using “mRMR” feature selection criterion. Support vector machines (SVMs) and extreme learning machines (ELMs) are compared for classification between clench speed and clench force motor imagery using the optimized feature. Our results show that no significant difference in the classification rate between SVMs and ELMs is found. The scaled feature combinations can get higher classification accuracy than the no-scaled feature combinations at significant level of 0.01, and the “mRMR” feature selection criterion can get higher classification rate than the “MIFS” feature selection criterion at significant level of 0.01. The time-frequency-phase feature can improve the classification rate by about 20% more than the time-frequency feature, and the best classification rate between clench speed motor imagery and clench force motor imagery is 92%. In conclusion, the motor parameter imagery paradigm has the potential to increase the direct control commands for BCI control and the time-frequency-phase feature has the ability to improve BCI classification accuracy.

## 1. Introduction

Brain computer interface (BCI) is an emerging technology in the last decades due to its ability to enable people to control devices using thought directly, such as computer cursors, robotic limbs, and prosthetic devices [1–3]. Furthermore, researchers have shown that the brain-to-brain interface (BBI) makes it possible for a human volunteer to control a rat's tail movement according to his/her intention [4].

Many cognitive tasks can modulate brain activities, such as motor imagery, mental calculation, and mental singing [5]. Brain activities caused by external stimulations can also be used for BCI, including steady state visual evoked potentials (SSVEP) [6] and P300 [7]. Among these approaches, motor imagery is widely used due to its convenience and no external stimulations [8]. Studies have shown that motor imagery presents similar brain activities as real movement [9].

The modulated brain signals for BCI control can be acquired by both invasive and noninvasive methods [10]. The mostly used noninvasive brain signal is electroencephalography (EEG) due to its high sampling frequency and low cost. The functional near-infrared spectroscopy (fNIRS) is another noninvasive approach to acquire cognitive related brain signals [11]. Also, EEG can be acquired with fNIRS simultaneously to obtain enhanced performance because the two approaches acquire cognitive related brain signals through both electrophysiology and hemodynamic aspect [12].

Before being used to control a device, the brain signals must be decoded first [13, 14]. Most researchers use time, spatial, and frequency features for mental decoding [15–19], but little attention is focused on phase feature. Lachaux et al. researched phase synchrony in brain signals using a statistical measure of phase locking value (PLV) for the first time in 1999 [20]. Gysels and Celka investigated phase synchronization for recognition of mental tasks for BCI in 2004, and their results showed that phase feature is useful for spontaneous EEG classification during mental tasks [21]. Li and Zhang compared PLV with phase interval value (PIV) for classification of motor imagery for BCI applications in 2009 and found that PIV performed better than PLV [22]. Some researchers also apply empirical mode decomposition (EMD) and multivariate extensions of empirical mode decomposition (MEMD) to obtain phase information for BCI and get good results [23]. However, MEMD is time consuming in situations of large channel number and high sampling frequency.

In this paper, we investigate the classification accuracy of different combinations of time-frequency feature and time-frequency-phase feature for motor parameter imagery classification using support vector machines (SVMs) and extreme learning machines (ELMs). Two feature selection criteria are also compared in the paper: the mutual information feature selection (MIFS) criterion and the max-relevance min-redundancy (mRMR) criterion. The Hilbert transform is applied on the mu and beta band EEG signal to get instantaneous phase (IP), as well as instantaneous amplitude (IA) and instantaneous frequency (IF). The band power (BP) feature is compared with these three features and their combinations. Our results show that IP performs best at a classification accuracy of 0.83 when used independently among these four feature types. The classification rate can be improved to 0.92 when the four feature types are used simultaneously. The performances of SVMs and ELMs are similar to each other, and the “mRMR” feature selection criterion performs better than the “MIFS” criterion.

The paper is constructed as follows. In Section 2, we will describe the experiment design in this research. Then, the data analysis methods are presented in Section 3. The results of data analysis are presented in Section 4. Finally, we will discuss the results of this research and make some conclusions in Section 5.

## 2. Experiment Design

### 2.1. Experiment Paradigm

**Figure 1:**
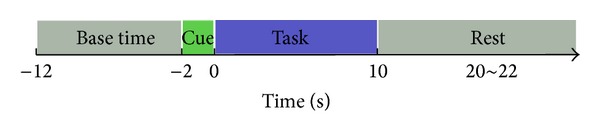
The experiment paradigm used in the research.

Traditional BCI paradigms use motor imagery of different limbs to modulate brain signals and can generally obtain at most 4 or 5 direct control commands [24, 25]. In our research, we adopt the motor parameters imagery paradigm (Figure 1). Three levels of clench speed motor imagery of the right hand and three levels of clench force motor imagery of the right hand are used in the experiment. Subjects exercise right hand clench movement at the speeds of 0.5 Hz, 1 Hz, and 2 Hz according to a metronome. The maximum clench force (MF) of every subject is measured, and then subjects practice to clench their right hand at the levels of 20%, 50%, and 80% MF. During the experiment, subjects are required to recall the feeling of real movements. In this paper, we only investigate the possibility of discrimination of clench speed motor imagery from clench force motor imagery. The reason for using three levels for clench speed and clench force motor imagery is to eliminate the effects of different task intensities. The advantage of our paradigm is the potential ability to provide more direct control commands for BCI applications.

We acquired EEG and fNIRS signals simultaneously. The analysis results of fNIRS signal have been presented in paper [26], and, in this paper, we only analyze the EEG signals. The analysis of enhanced BCI performance of EEG-fNIRS combined feature will be discussed in the future.

Taken into the consideration of time lag in fNIRS response to motor imagery [27], the duration of a single trial is much longer than traditional EEG paradigms. In our experiment, a single trial comprises four parts: 10-second base line period, 2-second cue period, 10-second task period, and 10~12-second rest period. Subjects are told not to blink their eyes during the motor imagery period. Every subject takes part in 3 sessions, and each session consists of 60 trials. To avoid subject fatigue, the trial number in a session is much less than traditional EEG paradigms. So we adopt the 5-fold cross-validation approach to reduce the effects of small trial number.

Six right handed healthy subjects (three males and three females, average age: 26.8 years) participate in the experiment. Three of them are trained three times before the experiment, and the others take part in no training course. All the subjects give written informed consent to participate in the experiment. Also, the experiment is approved by the Ethical Committee of the Shenyang Institute of Automation (SIA), Chinese Academy of Sciences (CAS).

### 2.2. Data Acquisition

**Figure 2:**
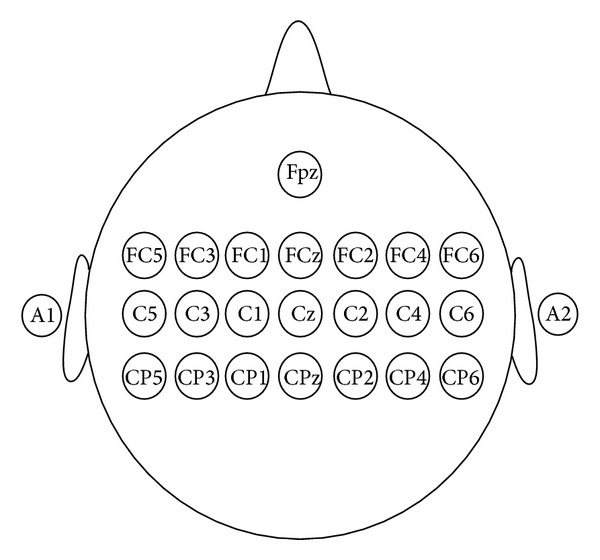
The electrodes used in the experiment.

21 Ag/AgCl electrodes above the primary motor cortex and the supplementary motor cortex are used in the experiment, as shown in Figure 2. A1 is used as the reference, and Fpz is used as the ground. Neuroscan synamps2 is used to acquire EEG signals from all the channels at a sampling frequency of 1000 Hz. The electrode impedance is reduced to 5 K Ohms before the experiment. The Electrooculogram (EOG) is also recorded to ensure that no EOG artifacts exist during the motor imagery task period.

## 3. Data Analysis Methods

The original EEG data are low-passed at a cutoff frequency of 125 Hz and then down sampled to 250 Hz to decrease the computation cost. Then, the frequencies from 5 Hz to 45 Hz are extracted for the following analysis. To improve the spatial resolution of the EEG data, a small Laplacian filter [28] is used as in

(1) $$\begin{array}{c} V_{j}^{\text{Lap}}=V_{j}-\frac{1}{N}\underset{k\in S_{j}}{\sum }V_{k}, \end{array}$$

where *V*_*j*_ is the *j*th channel, *S*_*j*_ is the surrounding channel set of *V*_*j*_, *N* is the size of *S*_*j*_, and *V*_*k*_ is the *k*th channel in *S*_*j*_.

As former researches show that mu rhythm and beta rhythm are effective for BCI control, the signals of 8–12 Hz and 18–25 Hz are extracted, respectively, for further analysis [29].

### 3.1. Hilbert Transform

Two methods can be used to get the phase information of a signal. The first one is the Hilbert transform method, and the other one is the complex wavelet convolution method [20]. Researches show that the results of the two methods are comparable [30]. We adopt the Hilbert transform method in the research.

The Hilbert transform of a signal *x*(*t*) can be gotten by convolution with the function *h*(*t*) = 1/*πt*,

(2) $$\begin{array}{c} y\left(t\right)=P\overset{\infty }{\underset{-\infty }{\int }}x\left(\tau \right)h\left(t-\tau \right)d\tau =\frac{1}{\pi }P\overset{\infty }{\underset{-\infty }{\int }}\frac{x\left(\tau \right)}{t-\tau }d\tau , \end{array}$$

where *P* is the Cauchy principal value. The analytic signal of *x*(*t*) can be gotten by (2). Consider

(3) $$\begin{array}{c} z\left(t\right)=x\left(t\right)+iy\left(t\right)=A\left(t\right)e^{i\theta (t)}, \end{array}$$

where *i* is the imaginary unit, *A*(*t*) = sqrt(*x*(*t*)^2^ + *y*(*t*)^2^) is the instantaneous amplitude (IA), and *θ*(*t*) = arctan*y*(*t*)/*x*(*t*) is the instantaneous phase (IP). The value range of *θ*(*t*) is $\left[\begin{array}{cc} -\pi & \pi \end{array}\right]$. The instantaneous frequency (IF) can be gotten by *w*(*t*) = *dθ*(*t*)/*d*(*t*). To get the correct instantaneous frequency, *θ*(*t*) must be unwrapped by adding multiples of ±2*π* when absolute jumps of more than *π* happen between consecutive elements.

### 3.2. Feature Extraction

Four feature types are researched in the paper: the power, the instantaneous amplitude (IA), the instantaneous phase (IP), and the instantaneous frequency (IF). The latter three features can be gotten from Hilbert transform.

To decrease the feature dimensions and improve the classification stability, the original features are averaged using an 0.5 s moving window with the step width of 0.125 s. Four window length and step width combinations are tested (0.5 s–0.125 s, 0.5 s–0.2 s, 1 s–0.125 s, and 1 s–0.2 s), and we find that the 0.5 s–0.125 s combination performs the best of all. The 0.5 s window length is reasonable taken into the consideration of the varied nature of EEG signals, and the 0.125 s step width is enough for fluent BCI device control. The averaged feature points between the time range of $\left[\begin{array}{cc} -0.5 & 0.5 \end{array}\right]$ of all the channels are combined into a vector, and the different feature vector types are normalized and merged into a single vector according to the feature type combination situations.

In our research, we compared four different feature types and four different feature combination types. The four combination types are power-phase combination, amplitude-phase combination, amplitude-phase-frequency combination, and power-amplitude-phase-frequency combination.

### 3.3. Normalization

Due to the value range of the four feature types differing significantly from each other, normalization is essential to eliminate the degradation of the classifier performance and get better classification accuracy using the merged feature. In our paper, the feature vector of different types is normalized to the range $\left[\begin{array}{cc} -1 & 1 \end{array}\right]$ using (4), and the normalized feature vectors are merged into a single vector for the following feature optimization and classification:

(4) $$\begin{array}{c} \text{Featur}{\text{e}}_{\mathrm{norm}}=\left(\frac{\text{Feature}-\min\left(\text{Feature}\right)}{\max\left(\text{Feature}\right)-\min\left(\text{Feature}\right)}-0.5\right)\times 2. \end{array}$$

### 3.4. Feature Optimization

The original feature space may contain much redundant information, which may reduce the classification accuracy significantly. Feature optimization is the key to improve the discriminative performance of a classifier. Principle component analysis (PCA) is one type of feature optimization techniques that project the original feature space to another one [31]. The disadvantage of PCA is that the converted feature space is hard to understand. Feature selection is another type of feature optimization methods. This method selects a subset of features from the original feature space according to some criteria. Depending on whether the classifier is included in the selection process, feature selection methods can be grouped into “wrapper” methods and “filter” methods [32]. Generally speaking, the “wrapper” methods take the classifier's classification accuracy as the feature selection criterion, thus getting better classification rate. However, its generalization ability is poor and its computational burden is much harder. In our research, we choose the “filter” method and compare two information based feature selection criteria: the mutual information feature selection (MIFS) criterion [33] and the max-relevance min-redundancy (mRMR) feature selection criterion [34].

The MIFS criterion uses the following equation to score the potentially usefulness of a feature or feature subset:

(5) $$\begin{array}{c} J_{\text{MIFS}}\left(X_{k}\right)=I\left(X_{k};Y\right)-\beta \underset{X_{j}\in S}{\sum }I\left(X_{k};X_{j}\right), \end{array}$$

where *I*(*X*_*k*_; *Y*) is the mutual information [35] between feature *X*_*k*_ and class label *Y*, which is used to ensure feature relevance; *I*(*X*_*k*_; *X*_*j*_) is the mutual information between feature *X*_*k*_ and features already selected in the currently selected feature set *S*, which is used as a penalty to enforce low correlations. The value of *β* depends on the independence level between *X*_*k*_ and *X*_*j*_. A zero value means a full belief of the independence relations, and a one value means a full belief of the dependence relations.

The mRMR criterion uses the following equation to score the potentially usefulness of a feature or feature subset:

(6) $$\begin{array}{c} J_{\text{mRMR}}\left(X_{k}\right)=I\left(X_{k};Y\right)-\frac{1}{\left|S\right|}\underset{X_{j}\in S}{\sum }I\left(X_{k};X_{j}\right). \end{array}$$

The difference between MIFS criterion and mRMR criterion is that the *β* value of MIFS is set by experience, while the *β* value of mRMR is set inversely proportional to the size of the current feature set. The comparison between MIFS and mRMR can be found in [32, 36].

### 3.5. Support Vector Machines

Support vector machines (SVMs) have been used in the classification applications extensively due to their surprising classification ability [37]. Although it is originally proposed for binary classification, it can also be applied in multiclass classification problems through approaches of one-against-all (OAA) and one-against-one (OAO) methods [38]. SVMs can also be extended to solve regression problems by the introduction of the epsilon-insensitive loss function [39, 40]. By mapping the input samples (vectors) into a higher dimensional feature space using a kernel function [41] and by selecting the samples (the support vectors) that can produce the largest margin between two classes, SVMs demonstrate amazing results for both linear classification problems and nonlinear classification problems.

The decision function of SVMs has the following form:

(7) $$\begin{array}{c} f\left(x\right)=\mathrm{sign}\left(\overset{N}{\underset{i=1}{\sum }}w_{i}t_{i}\phi \left(x,x_{i}\right)+b\right), \end{array}$$

where *x*_*i*_ is the training sample, *t*_*i*_ is the sample's target class label, *w*_*i*_ is the weight vector used as a normal vector to determine the classification hyperplane, *b* is a bias to adjust the location of the hyperplane for better classification results, and *ϕ*(*x*, *x*_*i*_) is the kernel function to map the sample into higher feature space. In our research, the linear kernel *K*(*x*_*i*_, *x*_*j*_) = *x*_*i*_^*T*^ *x*_*j*_ is used because it requires less parameters to optimize and can achieve much higher classification results compared with other kernels when their parameters are not optimized.

The distance between two different classes in the feature space is 1/||*w*||, so SVMs determine the classifier parameters by solving the following optimization problem:

(8) min⁡w,b,ξ 12wTw+C∑i=1Nξisubject  to yi(wTϕ(x,xi)+b)≥1−ξiξi≥0, i=1,…,N,

where *ξ*_*i*_ is the training error and *C* is a user-defined constant parameter used to provide a tradeoff between the separating margin and the training error. The optimization problem can be solved by Lagrange methods [37].

### 3.6. Extreme Learning Machines

Extreme learning machines (ELMs) are types of single-hidden layer feedforward neural networks (SLFNs) [42], which can be used for both regression and multiclass classification [43]. Unlike other feedforward neural networks that use gradient-based learning algorithms to tune all the network parameters iteratively, ELMs choose the input weights randomly and determine the output weights of SLFNs using an analytical approach. The advantages of ELMs contain extremely fast learning speed, the smallest training error, and better generalization performance. Huang et al. have rigorously proved that the input weights and hidden layer biases of SLFNs with infinitely differentiable activation functions can be randomly assigned [44].

For a training set *ℵ* = {(*x*_*i*_, *t*_*i*_)∣*x*_*i*_ ∈ *R* ^*n*^, *t*_*i*_ ∈ *R* ^*m*^, *i* = 1,…, *N*}, standard SLFNs with activation function *g*(*x*) and a hidden node number $\bar{N}$ are mathematically modeled as

(9) $$\begin{array}{c} \overset{\bar{N}}{\underset{i=1}{\sum }}\beta_{i}g_{i}\left(x_{j}\right)=\overset{\bar{N}}{\underset{i=1}{\sum }}\beta_{i}g\left(w_{i}\cdot x_{j}+b_{i}\right)=t_{j}\;j=1,\ldots ,N, \end{array}$$

where *w*_*i*_ is the input weight vector that connects the input nodes and the *i*th hidden node, *β*_*i*_ is the output weight vector that connects the *i*th hidden node and the output nodes, and *b*_*i*_ is the bias of the *i*th hidden node.

Equation (9) can be written in a compact format as

(10) $$\begin{array}{c} H\beta =T, \end{array}$$

where

(11) $$\begin{array}{c} H={\left[\begin{array}{ccc} g(w_{1}\cdot x_{1}+b_{1}) & \cdots & g(w_{1}\cdot x_{1}+b_{\bar{N}})\\ \vdots & \cdots & \vdots \\ g(w_{1}\cdot x_{N}+b_{1}) & \vdots & g(w_{\bar{N}}\cdot x_{N}+b_{\bar{N}}) \end{array}\right]}_{N\times \bar{N}},\\ \beta ={\left[\begin{array}{c} \beta_{1}^{T}\\ \vdots \\ \beta_{\bar{N}}^{T} \end{array}\right]}_{\bar{N}\times m},\;\;T={\left[\begin{array}{c} t_{1}^{T}\\ \vdots \\ t_{\bar{N}}^{T} \end{array}\right]}_{N\times m}. \end{array}$$

When the input weight *w*_*i*_ and the bias *b*_*i*_ are given, the hidden layer output matrix *H* can be calculated, and then the output weight *β* can be calculated using the following equation:

(12) $$\begin{array}{c} \beta =H^{†}T, \end{array}$$

where *H* ^†^ is the Moore-Penrose generalized inverse of matrix *H* [45].

### 3.7. Common Spatial Patterns

To validate the advantage of our method, we also classify clench speed and clench force motor imageries using the method of common spatial patterns (CSP). CSP is first applied to discriminate movement-related patterns by Müller-Gerking et al. in 1999 [46], and it has become a very popular method with many variants for motor imagery classification [47–49]. This method performs a weighting of the electrodes to maximize the difference between two different motor tasks, and the channel variance of the filtered signal is used for the classification in the following steps. The details of the algorithm can be found in [18].

In our research, the original EEG data are down sampled to 250 Hz first and then filtered in an 8–30 Hz band. No Laplacian filter is used to avoid zero eigenvalue when calculating the CSP model. Only two most important CSP patterns are used to get channel variance features; then SVMs and ELMs are used to classify these features, respectively.

## 4. Results

**Figure 3:**
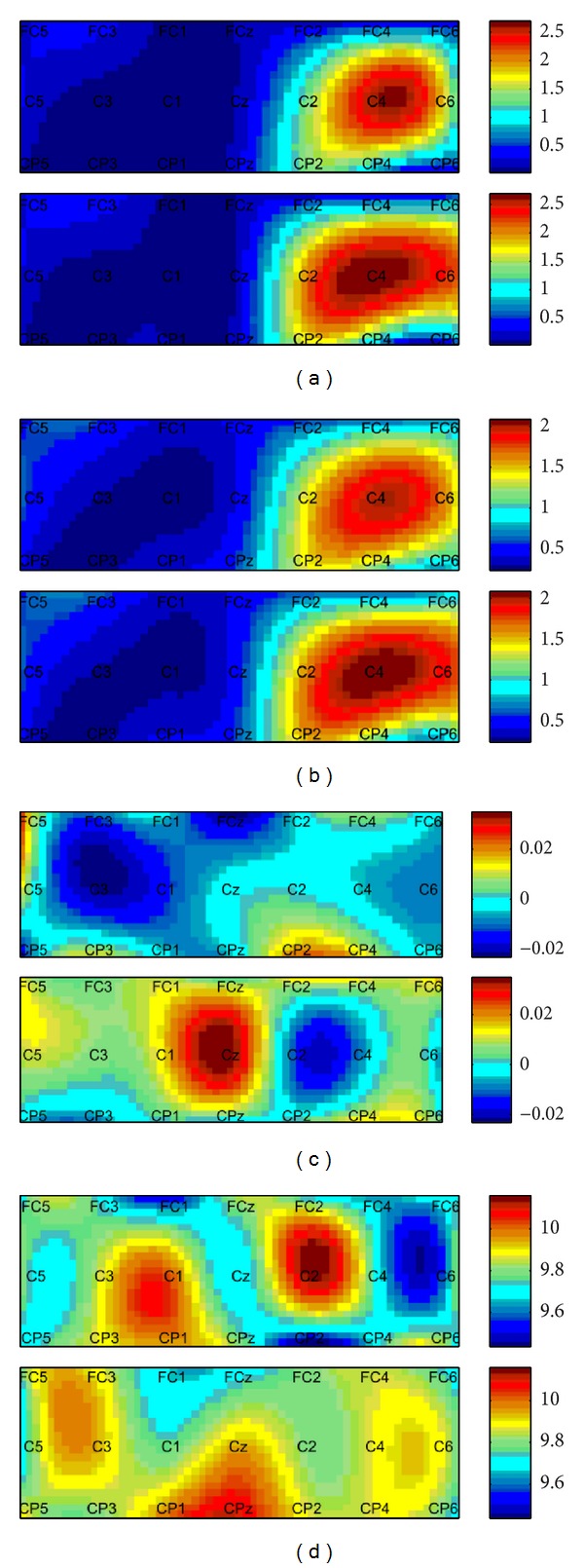
The topography of four different mu feature types of a subject session. The mean feature value during $\left[\begin{array}{cc} 0 & 0.5 \end{array}\right]$ period is used for the plot. (a) The topography of power. (b) The topography of the instantaneous amplitude (IA). (c) The topography of the instantaneous phase (IP). (d) The topography of the instantaneous frequency (IF).

The topographies of the four different feature types are shown in Figure 3. While topographies of power feature and IA feature show little difference between clench force motor imagery and clench speed motor imagery, the topographies of IP and IF feature show significant difference between the two motor imagery tasks, which means that phase feature and its derivative contain some different information compared to the amplitude feature and power feature for motor parameters imagery.

The classification results of SVMs and ELMs using three different feature extraction methods (no-scaled with MIFS feature selection criterion, scaled with MIFS feature selection criterion, and scaled with mRMR feature selection criterion) and eight different feature types/combinations are shown in Table 1 and Figure 5. No significant difference between the classification rates between SVMs and ELMs for all the conditions can be found at the confidence level of 0.01 using *t*-test.

**Figure 4:**
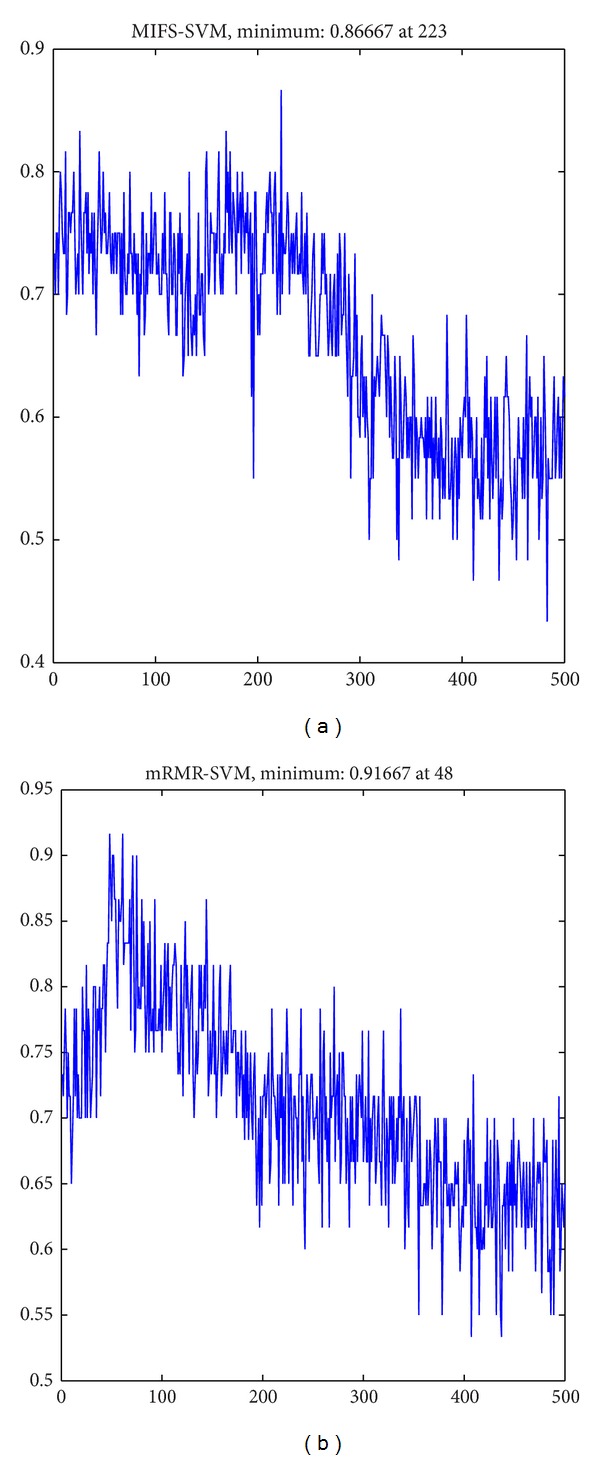
The comparison of classification accuracy using different feature selection criteria. (a) The classification accuracy using a different number of features selected by MIFS feature selection criterion. (b) The classification accuracy using a different number of features selected by mRMR feature selection criterion.

**Figure 5:**
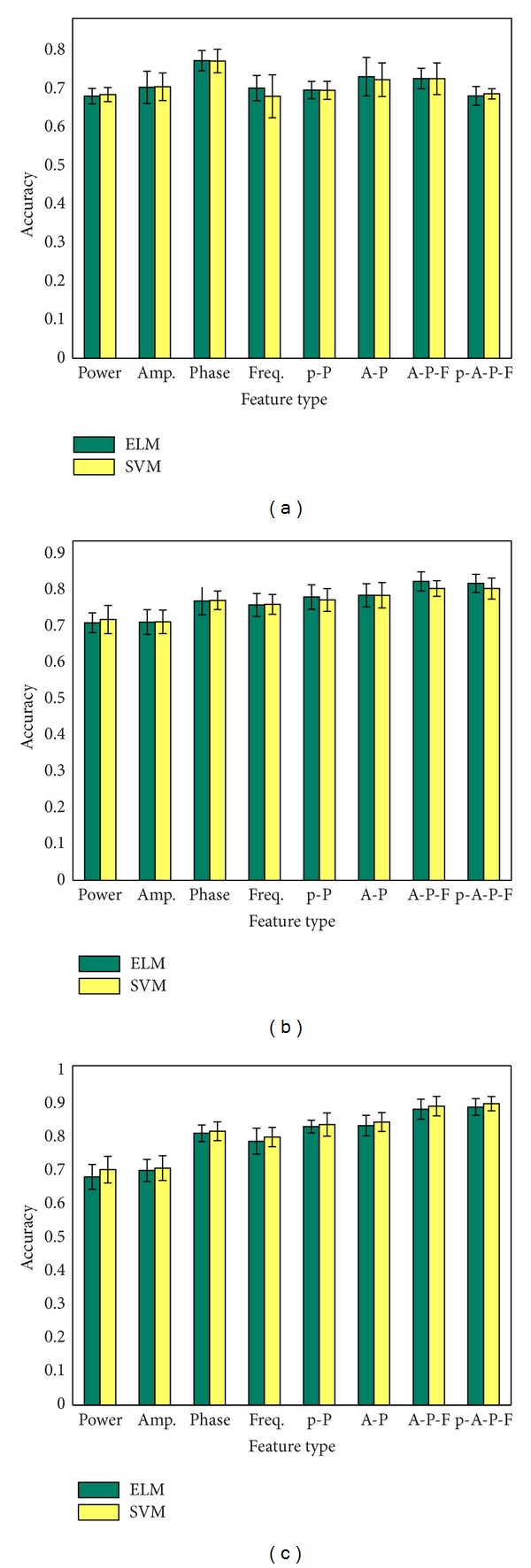
Comparison between the classification results between ELMs and SVMs. (a) Using the original feature and MIFS feature selection criterion. (b) Using the scaled feature and MIFS feature selection criterion. (c) Using the scaled feature and mRMR feature selection criterion.

**Figure 6:**
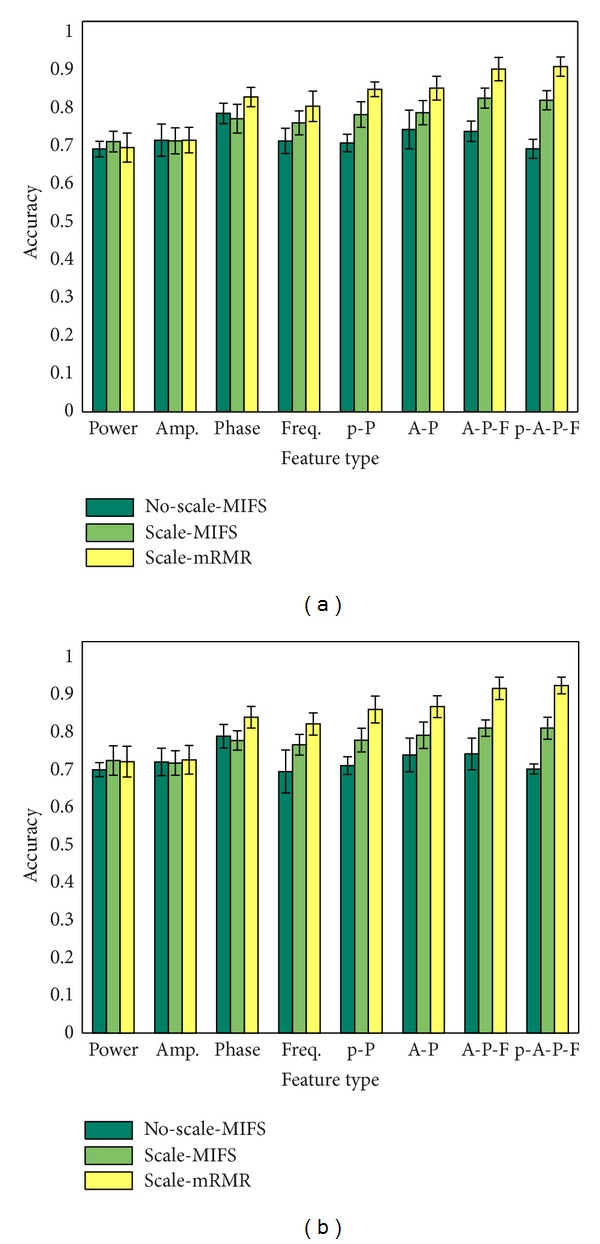
The comparison of three different feature extraction methods: the first one is no-scaled feature chosen by MIFS feature selection criterion; the second one is scaled feature chosen by MIFS feature selection criterion; the last one is scale feature chosen by mRMR feature selection criterion. (a) The comparison using ELMs. (b) The comparison using SVMs.

Generally speaking, the scaled features have higher classification accuracy than the no-scaled features, and the mRMR feature selection criterion has higher classification accuracy than the MIFS feature selection criterion, as shown in Figures 4 and 6 and Tables 2 and 3. The feature numbers selected in the best feature subset using MIFS and mRMR are 64 ± 83 and 114 ± 74 for SVMs and 115 ± 66 and 107 ± 102 for ELMs. The classification accuracy of power feature and IA feature shows no significant difference. For the results using mRMR feature selection criterion and SVMs classifier, the IP feature and IF feature both have higher classification accuracy than power and IA feature at the confidence level of 0.01; both “IA-IP-IF” and “power-IA-IP-IF” feature combinations have higher classification accuracy than the other 6 features or feature combinations at a confidence level of 0.01. No significant difference between these two combinations is found.

The best classification rate between clench speed motor imagery and clench force motor imagery is 92% when “power-IA-IP-IF” combination feature and SVMs classifier are used. For comparison, the classification accuracies using CSP are 0.73 ± 0.03 and 0.75 ± 0.03 using ELMs and SVMs, respectively. This result demonstrates not only that using motor parameters imagery for BCI applications is possible but also that the time-frequency-phase method outperforms traditional CSP method.

## 5. Discussions and Conclusions

In this paper, we present the usefulness of phase information for BCI applications, which has been researched by few researchers before. We also demonstrate a new motor parameter imagery paradigm using clench speed and clench force as imagery tasks, and the results show that this paradigm has the potential ability to provide more direct control commands for BCI systems.

Two popular classification methods are compared in the paper using 5-fold cross validation, and no significant difference in the results is found between them. We only use the linear kernel for SVMs in this paper, and other kernels with parameter optimization may get better results. However, both kernel mapping and parameter optimization process require much computation cost. On the other hand, ELMs calculation is much simpler and faster, and no optimization process is needed, which is convenient for more applications.

We compare four different feature types (power, instantaneous amplitude [IA], instantaneous phase [IP], and instantaneous frequency [IF]) and four different feature combinations (power-IP, IA-IP, IA-IP-IF, and power-IA-IP-IF). The IA, IP, and IF features are calculated by Hilbert transform. We should notice that the IF feature is the derivative of the IP feature.

Three feature optimization processes are compared in this research: no-scaled feature using MIFS feature selection criterion, scaled feature using MIFS feature selection criterion, and scaled feature using mRMR feature selection criterion. Generally speaking, scaled feature combinations have higher classification accuracy than no-scaled feature combinations at a significant level of 0.01, which means that normalization is essential when merging two or more features together. The comparison of classification accuracy between MIFS feature selection criterion and mRMR feature selection criterion in Figure 4 demonstrates that the original feature space contains much redundant and irrelevant information for classification, and the mRMR criterion can choose the best feature subset more efficiently. The comparison of these two feature selection criteria can also be found in [32, 36]. The numbers selected in the best feature subset vary between different subjects and different sessions due to the variability of EEG signals, which means that the best feature indices should be adjusted every time for online applications.

When the four feature types are used independently, the IP feature and the IF feature both have higher classification accuracy than the power feature and the IA feature, but no significant difference is found between the results of IP and IF feature. When features are used in combinations, “IA-IP-IF” and “power-IA-IP-IF” feature combinations get the highest classification rate compared to the other 6 features/feature combinations. No significant difference is found between these two feature combinations, which means that the “IA-IP-IF” feature combination is enough for BCI applications.

In our research, all the features are extracted during the time range of $\left[\begin{array}{cc} -0.5 & 0.5 \end{array}\right]$, so our feature space contains feature of time domain. The four feature types are extracted from the mu rhythm and the beta rhythm, so the feature space contains feature of frequency domain. The IP feature and the IF feature are phase domain. So the “IA-IP-IF” and “power-IA-IP-IF” feature combinations are features of time-frequency-phase domain. Our results show that the usage of time-frequency-phase feature can improve the classification accuracy by about 20% and 15% compared to the time-frequency feature and the CSP method, respectively, which is very useful for improving BCI accuracy. Amplitude and phase are two important characteristics to describe a signal precisely. So, the time-frequency-phase feature can extract more information embedded in the motor imagery related EEG signals than the time-frequency features. Also, our results show that the classification between clench speed motor imagery and clench force motor imagery is possible, and the motor parameters imagery paradigm has the potential to increase the direct control commands for BCI applications.

In the future, we will analyze the EEG feature and the fNIRS feature simultaneously and investigate whether the multimodality method can improve the classification accuracy for BCI systems.

## Figures and Tables

**Table 1:** The classification results using different feature processing methods and different feature types.

	Power	IA	IP	IF	Power-IP	IA-IP	IA-IP-IF	Power-IA-IP-IF
No-scaled-MIFS								
ELMs	0.69 ± 0.02	0.71 ± 0.04	0.78 ± 0.03	0.71 ± 0.03	0.71 ± 0.02	0.74 ± 0.05	0.74 ± 0.03	0.69 ± 0.02
SVMs	0.69 ± 0.02	0.71 ± 0.04	0.78 ± 0.03	0.69 ± 0.06	0.71 ± 0.02	0.73 ± 0.04	0.74 ± 0.04	0.70 ± 0.01
Scaled-MIFS								
ELMs	0.71 ± 0.03	0.71 ± 0.03	0.77 ± 0.04	0.76 ± 0.03	0.78 ± 0.03	0.79 ± 0.03	0.82 ± 0.03	0.82 ± 0.03
SVMs	0.72 ± 0.04	0.71 ± 0.03	0.77 ± 0.03	0.76 ± 0.03	0.77 ± 0.03	0.79 ± 0.03	0.80 ± 0.02	0.80 ± 0.03
Scaled-mRMR								
ELMs	0.69 ± 0.04	0.71 ± 0.03	0.83 ± 0.03	0.80 ± 0.04	0.85 ± 0.02	0.85 ± 0.03	0.90 ± 0.03	0.91 ± 0.03
SVMs	0.72 ± 0.04	0.72 ± 0.04	0.83 ± 0.03	0.82 ± 0.03	0.85 ± 0.04	0.86 ± 0.03	0.91 ± 0.03	0.92 ± 0.02

**Table 2:** The *t*-test comparison between different feature processing methods using ELMs (the confidence level is 0.01).

Conditions	Power	IA	IP	IF	Power-IP	IA-IP	IA-IP-IF	Power-IA-IP-IF
No-scale-MIFS < scale-MIFS	0	0	0	0	1	0	1	1
Scale-MIFS < scale-mRMR	0	0	1	0	1	1	1	1
No-scale-MIFS < scale-mRMR	0	0	1	1	1	1	1	1

**Table 3:** The *t*-test comparison between different feature processing methods using SVMs (the confidence level is 0.01).

Conditions	Power	IA	IP	IF	Power-IP	IA-IP	IA-IP-IF	Power-IA-IP-IF
No-scale-MIFS < scale-MIFS	0	0	0	0	1	0	1	1
Scale-MIFS < scale-mRMR	0	0	1	1	1	1	1	1
No-scale-MIFS < scale-mRMR	0	0	1	1	1	1	1	1
